# Warning: This may be as dangerous as firearm injuries;“grease-gun injury”: A case report

**DOI:** 10.11604/pamj.2015.20.40.5892

**Published:** 2015-01-14

**Authors:** Oguz Eroglu, Elif Sari, Sevilay Vural, Figen Coskun

**Affiliations:** 1 Department of Emergency Medicine, Kirikkale University, Faculty of Medicine, Kirikkale, Turkey; 2 Department of Plastic and Reconstructive Surgery, Kirikkale University, Faculty of Medicine, Kirikkale, Turkey; 3 Department of Emergency Medicine Bartin State Hospital, Bartin, Turkey

**Keywords:** Grease-gun, firearm injuries, amputation

## Abstract

High-pressure injection gun (Grease-gun) injuries mainly occur with industrial labourers. Injuries associated with high pressure grease guns are very rare and frequently involve the hand and chest. The non-dominant hand is generally injured since the grease gun is usually held in the dominant hand. Even if high-pressure injection injury causes only a small lesion in the skin, it is still characterized by severe damage to subcutaneous tissue. Since initial presentation may be deceptive, treatment is frequently delayed. The characteristics of the material injected need to be known as a priority, and systemic intoxication must be ruled out. The risk of amputation is 16-55%. With solvents it goes up to 50-80%. Surgical treatment must be performed immediately, under general anesthesia or plexus block. Foreign material and necrotic tissue must be early debrided with wide microsurgical exploration. Positive outcomes in reacquisition of hand functions can be obtained with long-term and early physiotherapy.

## Introduction

Injuries associated with high pressure grease guns are very rare. Cases encountered in clinical practice frequently involve injury to the hand and chest, although they may, rarely, be seen in other regions of the body. Rapid treatment is very important in injuries causing high suspicion and circulatory compromise. The purpose of this case report is to emphasize the importance of injuries associated with injection guns, which we believe is not sufficiently appreciated with the naked eye.

## Patient and observation

**Figure 1:**
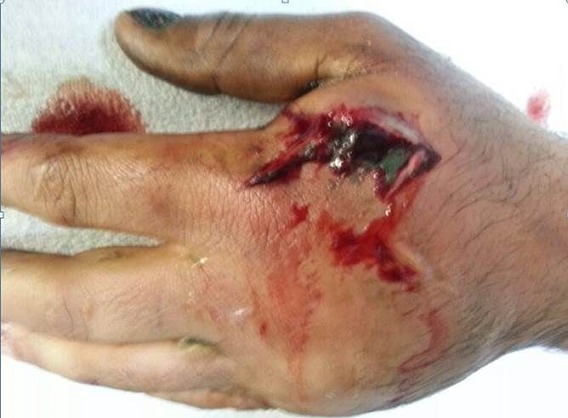
Pictures of patient's hand after the trauma

**Figure 2:**
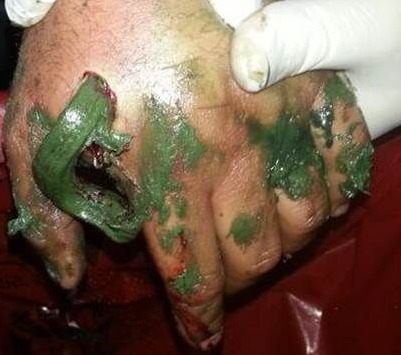
Pictures of patient's hand after the trauma

**Figure 3:**
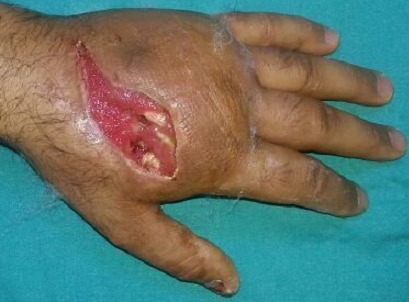
Pictures of patient's hand after the treatment

**Figure 4:**
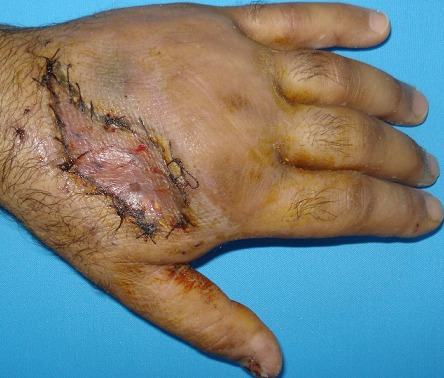
Pictures of patient's hand after the treatment

A 33-year-old man was brought to the emergency department with a work-related injury. A cutaneous injury with irregular margins, approximately 4 cm long, was present at the 2nd metacarpal level on the dorsal surface of the (Right hand dominant) patient's left hand (Figure 1, Figure 2). In addition to widespread tissue swelling, capillary refill time was decreased. Sensory examination was normal, while at motor examination movements were free in all directions, but painful. Fentanyl was administered IV for analgesia. Accumulated grease was observed in the subcutaneous tissue, part of which protruded from the wound. We tried to gently remove the grease from the tissue without applying extreme pressure. No fracture was observed in bone tissue at radiographic imaging, although there was an accumulation of material in the subcutaneous tissue. The patient was taken for surgery after tetanus and antibiotic prophylaxis. Following gradual debridement and washing of the wound site, hand physiotherapy was started. Wound granulation improved, and the defect in the dorsal aspect of the hand was repaired usingsplit thickness skin graft from the lateral aspect of the thigh. No problems were observed postoperatively(Figure 3, Figure 4).

## Discussion

High pressure “grease guns”are used in the maintenance of heavy industrial equipment. Accidents during their use may involve grease being injected into the body. Injuries are rare and frequently involve the hand and chest. Grease gun injuries represent approximately 1 in 600 hand injuries. Major hand surgery centers treat 1-4 grease gun injuries a year [1–3]. The non-dominant hand is generally injured since the grease gun is usually held in the dominant hand. The index finger is affected in more than 50% of cases. The palm and other regions of the hand are less commonly involved [4]. Injuries generally occur in males. Mean age observed is 36. Injuries usually involve the non-dominant hand and occur while cleaning the device or due to a leak from one of the tubes [5]. The entry hole is usually deceptively easily visible in grease gun injuries, being several millimeters in diameter. However, the grease frequently progresses along the skin and subcutaneous tissue as far as the deep muscle tissue [2, 3]. Grease gun-associated injuries must always be treated promptly. Early surgical intervention must be performed in high-risk situations, when the grease is suspected to have traveled some distance from the entry hole or when major vascular injury is expected in the region of the injury [6, 7]. The grease gun's pressure level plays an important role in the pathophysiology of injury. Grease guns employ a pressure of 40-800 bar/sec. A pressure of 7 bars is sufficient to cause damage to skin and subcutaneous tissue [8, 9]. The material injected may spread along the neurovascular bundle due to the low resistance in subcutaneous tissue. Traumatic dissection may occur in the fingers as a result; this may also lead to contraction of neurovascular structures, vascular spasm, tissue ischemia and thrombosis. The amount of fluid material is another factor determining tissue damage. This determines the extent of tissue tension and edema and may lead to compartment syndrome by compromising tissue perfusion. Destruction in tissue and cells and inflammatory response or systemic toxicity may develop in association with the chemical characteristics of the fluid material. Information concerning the chemical properties of the material injected and about an antidote if one is available should be obtained from a poison information center.

Finally, ischemia and destruction in tissue increases the risk of infection. Patients must therefore be started on antibiotics effective against gram (+) and gram (-) bacteria. Use of corticosteroids is controversial, but does not affect the incidence of amputation. Paints and solvents are more irritating substances than grease and are more lethal to cells [4, 10, 11]. Presence of grease causes a severe inflammatory response in tissue, and may also lead to significant systemic complications. Such injuries may have very serious consequences due to the tamponade effect of the material injected and the chemical damage caused by it [12]. Even if urgent and appropriate treatment is administered after grease gun injury, the results are still generally disappointing. The risk of amputation is 16-55%. The equivalent level for solvents is 50-80%. If blood supply compromise is present at first medical intervention the probable injury pressure is greater than490 bars, and the risk of amputation is 100% [6, 13]. Even if amputation is not required, permanent complications such as long-term contracture, loss of hand functions, decreased sensitivity, sold intolerance and hyperesthesia may ensue [4]. CT and MR are used to determine depth of trauma at diagnosis. MR is particularly useful in assessing the amount of grease remaining in tissue after surgery [1, 2, 14]. Systemic intoxication associated with the material involved must be ruled out in treatment. A poison information center should be consulted regarding the material involved and the antidote, if any. Vital parameters must be monitored. The physician must be prepared for kidney failure, allergic reaction or hemolysis. In particular, turpentine and white spirit entering the subcutaneous tissue from grease guns may give rise to severe intoxication [6]. Many authors recommend wide and rapid exploration under general anesthesia or plexus block as the most appropriate form of treatment. Merely squeezing the material out or enlarging the incision hole for purposes of providing relief and protecting the tissue is insufficient. All injected materials and necrotic tissues must be debrided and the area washed with saline solution. A splint must be applied postoperatively and the patient must immediately be started on physiotherapy. A program involving passive and active exercises must be provided [6, 13]. It is exceedingly important to apply preventive measures. Safety training must be provided for staff, and information must be given concerning use of protective gloves and clothing, as well as the potential severity of high pressure injection injuries [15].

## Conclusion

Even if high-pressure injection injury causes only a small lesion in the skin, it is still characterized by severe damage to subcutaneous tissue. Since initial presentation may be deceptive, treatment is frequently delayed. The characteristics of the material injected need to be known as a priority, and systemic intoxication must be ruled out. Surgical treatment must be performed immediately, under general anesthesia or plexus block. Foreign material and necrotic tissue must be debrided with wide microsurgical exploration. Positive outcomes in reacquisition of hand functions can be obtained with long-term and early physiotherapy. Personnel must be made aware of the severity and how to prevent grease gun injuries, and the requisite precautions should be taken.
